# Identification of Plasma-Generated Reactive Species in Water and Their DNA-Damaging Effects on Plasmid and Lymphocyte DNA

**DOI:** 10.3390/ijms26199385

**Published:** 2025-09-25

**Authors:** Stanislav Kyzek, Sára Pišteková, Ivana Kyzeková, Andrea Ševčovičová, Dušan Kováčik, Anna Zahoranová, Eliška Gálová

**Affiliations:** 1 Department of Genetics, Faculty of Natural Sciences, Comenius University Bratislava, Ilkovičova 6, Mlynská dolina, 842 15 Bratislava, Slovakia; pistekova7@uniba.sk (S.P.); ivana.kyzekova@uniba.sk (I.K.); andrea.sevcovicova@uniba.sk (A.Š.); eliska.galova@uniba.sk (E.G.); 2 Department of Experimental Physics, Faculty of Mathematics, Physics and Informatics, Comenius University Bratislava, Mlynská dolina, 842 48 Bratislava, Slovakia; dusan.kovacik@fmph.uniba.sk (D.K.); anna.zahoranova@fmph.uniba.sk (A.Z.)

**Keywords:** DNA damage, lymphocytes, non-thermal plasma, plasmid DNA, reactive oxygen and nitrogen species

## Abstract

Non-thermal plasma has attracted strong interest in medicine and agriculture due to its ability to generate reactive oxygen and nitrogen species (RONS). These species can stimulate wound healing and seed germination, but at higher levels they induce DNA damage—useful in cancer therapy but harmful when healthy cells must be preserved. Direct study of DNA damage in cells is difficult because of repair processes and protective barriers. To address this, we applied a dual-model system combining plasmid DNA and human lymphocytes exposed to plasma from the RPS40 device. Using selective scavengers, we identified hydroxyl radicals, ozone, and reactive nitrogen species as key mediators of DNA strand breaks and structural changes. Our results support a mechanistic model in which long-lived plasma-derived species (NOx, ozone, acids) dissolve in water and subsequently generate short-lived radicals such as hydroxyl radicals and peroxynitrite. These reactive molecules then directly attack DNA. This integrated approach—linking plasmid and cellular assays with scavenger-based identification of RONS—offers a novel and cost-effective method for dissecting plasma–DNA interactions. The findings provide mechanistic insight into how plasma-activated water damages DNA, guiding the safer and more effective application of plasma technologies in biomedical and agricultural contexts.

## 1. Introduction

Non-thermal plasma (NTP) is a partially ionized gas that fulfills defined criteria as conditions of quasi-neutrality, non-isothermality and others. In laboratory, plasma is usually generated using the electrical discharges after applying high electrical voltage to the gas. The strong electric field induces the process of molecules ionization due to which the electrons and ions accumulate. Due to the strong electric field effect, the electrons acquire a high energy corresponding temperature of the order of 10^4^ K and as the result of their collisions with molecules, chemically active particles such as free radicals, excited particles, neutral molecules including hydrogen peroxide, and temporary fields (i.e., heat, acoustic waves, electromagnetic fields) are formed. During plasma generation, the temperature of the working gas does not increase significantly, so the gas molecules are not in thermal equilibrium with the electrons (thermal non-equilibrium or non-isothermal plasma), and the processed material is not thermally damaged [1,2,3].

Since the temperature of NTP treatment can span from 30 to 40 °C, it is applicable to living cells, tissues, and other heat-sensitive material. Biological material can be treated with NTP directly or indirectly using plasma activated water (PAW) or other solutions and media. When water is treated by NTP, various reactions take place in the liquid, at the interface between liquid and air (gas) and in the working gas itself, in which the plasma is generated. More stable molecules dissolve in water and can participate in further reactions [4]. NTP also emits UV radiation that can cause the photolysis of molecules and ions such as hydrogen peroxide and nitrites [5,6]. Generally, water can be treated with NTP in three different ways: (A) direct contact of the plasma discharge with the liquid, (B) discharges in the gas phase of the plasma above the liquid (including cases where the electrode is the conducting liquid itself), and (C) plasma discharges in multiphase environments (e.g., in bubbles inside the liquids or contact with liquid sprays and foams) [4]. Reactive species present in NTP or PAW can induce oxidative stress in a cell or an organism, a condition resulting from the accumulation of reactive oxygen (ROS) or nitrogen species (RNS) due to an imbalance between their production and antioxidant-mediated elimination. When this balance is disturbed, cellular material is oxidized, and DNA could be damaged [7].

DNA damage induced by NTP treatment has been extensively studied [8,9,10,11]. Prolonged treatment times are commonly associated with higher amounts of DNA damage; however, the results may vary due to several factors such as the type of biological subject, plasma source set up, and working gas.

Similarly to experiments performed on cells or organisms, it has been shown that higher dose of plasma results in increasing incidence of both single-strand (SSBs) and double-strand breaks (DSBs) in plasmid DNA. Consensus amongst several authors seems to be that plasma causes mostly SSBs rather than DSBs [12,13,14]. However, Li et al. [15] observed that higher power output resulted in more intense formation of DSBs. It is possible that ROS interact with phosphodiester backbone of one strand and DSBs are likely caused by occurrence of two SSBs in proximity with each other [16,17]. Overall, DNA damage appears to be caused mainly by reactive molecules generated in plasma during treatment while UV has a smaller impact on formation of strand breaks [13,15]. However, which ROS are responsible for observed DNA damage is still not fully understood.

Some studies that employed scavengers that eliminate effects of specific ROS have seen a significant decrease in damages of both cellular components and DNA. Joshi et al. [8] found that non-thermal plasma generated by floating-electrode DBD in air caused oxidative DNA damage, lipid peroxidation, and disruption of membrane potential in *Escherichia coli*. This damage significantly decreased when certain ROS scavengers were added to PBS in which *E. coli* were treated by plasma. Therefore, authors contributed observed damages to singlet oxygen, hydrogen peroxide, ozone, and other ROS based on the scavenging activity of added solutions [8].

Despite this topic being heavily studied, it is not exactly known which of the reactive species are directly responsible for the cytotoxic and genotoxic effects in this scenario. This information is crucial for many potential biological applications of NTP, particularly in the biomedical field, where it has been investigated, i.e., for reducing tumor growth [18], enhancing sensitivity to oncotherapy [19], promoting wound healing and tissue regeneration [20], disinfecting root canals in dentistry [21], and treating skin infections [22]. Since these applications involve direct contact with living tissue, understanding the mechanisms of DNA damage, especially in human lymphocytes, is essential for evaluating both safety and therapeutic potential. Therefore, the aim of our study was to investigate the mechanism of plasma action on the water treated by Robust Plasma System 40 (RPS40) as well as estimate specific ROS and RNS, which may be present in the water and could damage DNA. A simple method, supplemented with various scavengers, was chosen to analyze the impact of individual ROS and RNS on a selected target of interest: plasmid DNA. Plasmid DNA was selected as a model system because it allows the direct assessment of ROS and RNS effects on DNA without interference from cellular repair mechanisms, chromatin packaging, or secondary reactive species. To further supplement these experiments, DNA damage in lymphocytes was investigated to compare the effects of NTP within and outside the cellular environment. Lymphocytes were chosen as a model system because they circulate throughout the body, making them a systemic indicator of genotoxic stress, and they are also a well-established cell type for comet assay applications. In these cells, DNA damage arises not only from direct exposure to plasma-derived species but also from intracellular metabolic processes, which were assessed using the comet assay. This assay is a simple yet powerful method for quantifying DNA damage in cellular systems such as lymphocytes, and it has been widely used in studies evaluating NTP-induced DNA damage in various types of cells [10,23,24,25,26,27,28]. Blackert et al. [23] used N-acetylcysteine in their study as a scavenger for hydrogen peroxide; however, the study did not provide results for changes in DNA damage after application of N-acetylcysteine to cells before NTP treatment. As far as we are aware, scavengers are not used often in this case to observe changes in the amount of DNA damage based on elimination of a particular ROS and RNS. Our work provides insight into this specific application of scavengers to comet assay and shows its disadvantages, while highlighting the use of plasmid DNA at the same time.

Naturally, the results obtained from the analyses on plasmid DNA cannot be directly extrapolated to living cells. Nevertheless, such a design of experiments represents the simplest system in which we can detect specific ROS and RNS in PAW that have a negative effect on naked DNA.

## 2. Results

### 2.1. Effects of NTP on Plasmid DNA in the Presence of Specific Scavengers

**Figure 1:**
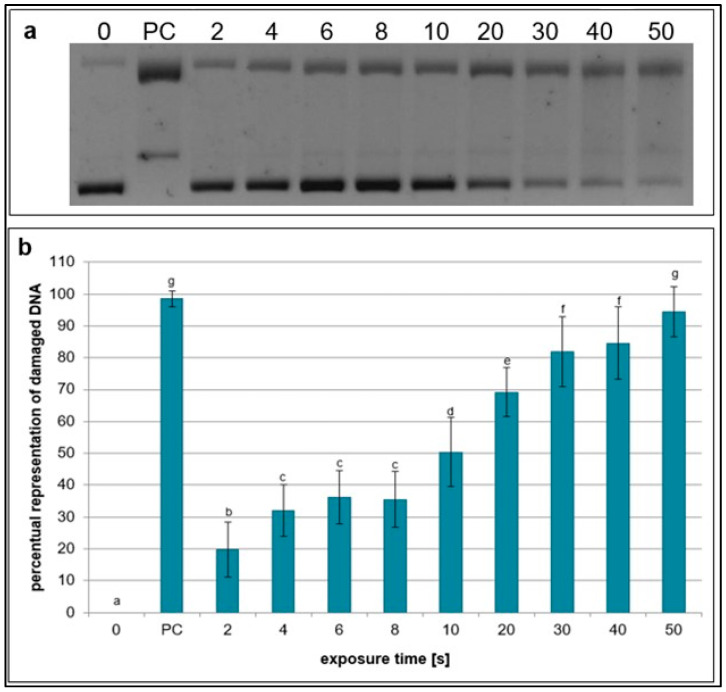
Representative gel (**a**) and graphical representation (**b**) of percentage of damaged DNA in plasmid samples exposed to NTP generated for 2 to 50 s using RPS40 plasma source assessed by DNA topology assay. PC (positive control—0.125 mM FeSO_4_.7H_2_O), 0 (negative control—sample without NTP exposure). Damaged DNA represents the amount of both SSBs and DSBs. Data are presented as mean ± SD. Statistically significant differences in the amounts of damaged DNA were determined using LSD ANOVA test at the significance level *p* ≤ 0.05. The significant difference between homogenous groups is depicted by a complete variation in the letters. The data are the results of twelve independent experiments.

The effects of NTP on break formation in plasmid DNA were analyzed using DNA topology assay. After the treatment with RPS40 plasma source, SSBs started to form even in the shortest exposure time (2 s) and their amount increased in a dose-dependent manner. The turning point, in which damaged DNA began to prevail over undamaged, was observed at 20 s exposure time (Figure 1).

**Figure 2:**
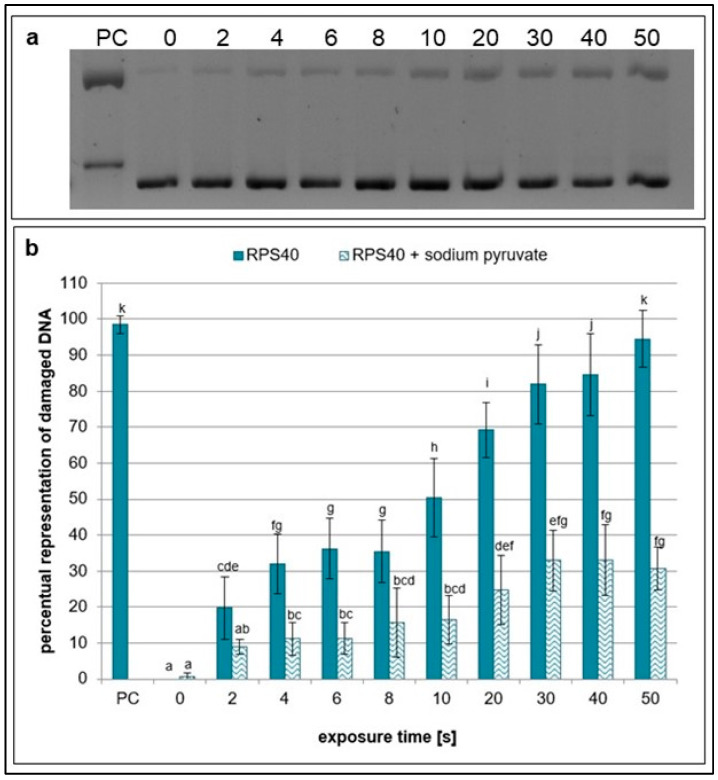
Representative gel (**a**) and graphical representation (**b**) of the percentage of damaged DNA in plasmid samples with sodium pyruvate (RPS40 + sodium pyruvate) and without a scavenger (RPS40) exposed to NTP for 2 to 50 s using RPS40 plasma source assessed by DNA topology assay. PC (positive control—0.125 mM FeSO_4_.7H_2_O), 0 (negative control—sample without NTP exposure). Damaged DNA represents the amount of both SSBs and DSBs. Data are presented as mean ± SD. Statistically significant differences in amounts of damaged DNA were determined using LSD ANOVA test at a significance level of *p* ≤ 0.05. The significant difference between homogenous groups is depicted by a complete variation in the letters. The data are the results of four independent experiments.

Subsequently, the formation of SSBs and DSBs in plasmid DNA after the addition of specific ROS and RNS scavengers to the reaction mixture before NTP treatment was analyzed. The scavenging effect of sodium pyruvate, which eliminates hydrogen peroxide [29], decreased with increasing exposure time. Additionally, a higher amount of undamaged DNA was present in the samples with sodium pyruvate compared to the samples without the scavenger at all treatment times, which indicates formation of hydrogen peroxide in the NTP-treated samples (Figure 2).

**Figure 3:**
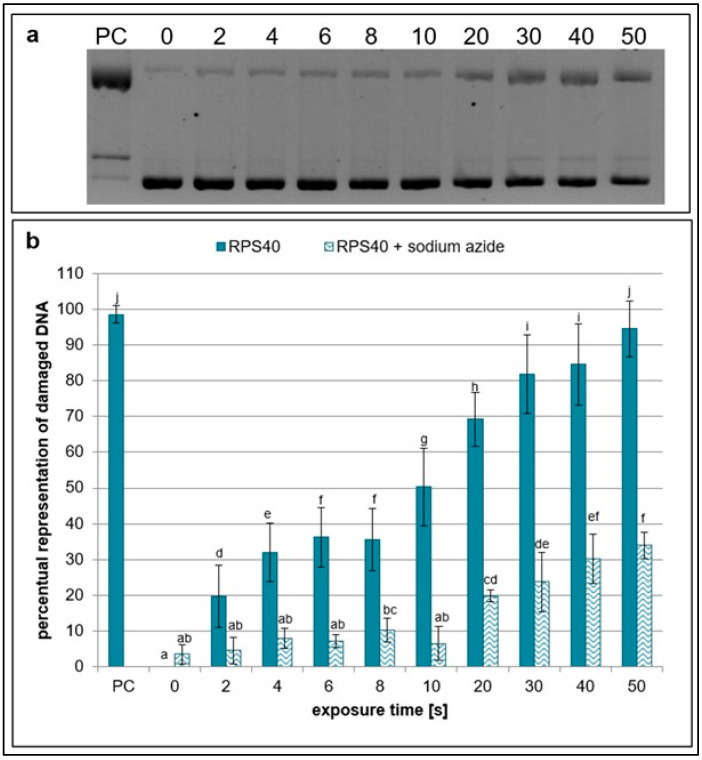
Representative gel (**a**) and graphical representation (**b**) of the percentage of damaged DNA in plasmid samples with sodium azide (RPS40 + sodium azide) and without a scavenger (RPS40) exposed to NTP for 2 to 50 s using RPS40 plasma source assessed by DNA topology assay. PC (positive control—0.125 mM FeSO_4_.7H_2_O), 0 (negative control—sample without NTP exposure). Damaged DNA represents the amount of both SSBs and DSBs. Data are presented as mean ± SD. Statistically significant differences in amounts of damaged DNA were determined using LSD ANOVA test at a significance level of *p* ≤ 0.05. The significant difference between homogenous groups is depicted by a complete variation in the letters. The data are the results of four independent experiments.

The scavenging activity of sodium azide, which targets singlet oxygen, hydroxyl radicals, and nitrites [29,30,31], decreases with increasing exposure time, similarly to sodium pyruvate, as evidenced by the increased amount of damaged plasmid DNA. Nonetheless, it had a stronger scavenging effect than sodium pyruvate at all exposure times as the amount of damaged DNA decreased more after the addition of sodium azide to the samples (Figure 3). These results indicate that NTP generated by RPS40 induces the formation of hydroxyl radicals, nitrites, and singlet oxygen, which are significantly scavenged by sodium azide.

**Figure 4:**
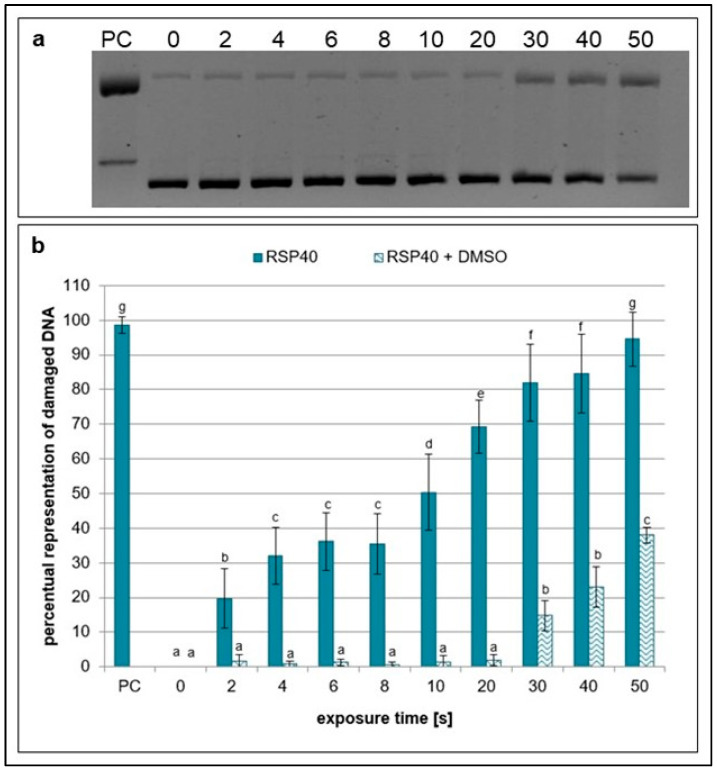
Representative gel (**a**) and graphical representation (**b**) of the percentage of damaged DNA in plasmid samples with DMSO (RPS40 + DMSO) and without a scavenger (RPS40) exposed to NTP for 2 to 50 s using RPS40 plasma source assessed by DNA topology assay. PC (positive control—0.125 mM FeSO_4_.7H_2_O), 0 (negative control—sample without NTP exposure). Damaged DNA represents the amount of both SSBs and DSBs. Data are presented as mean ± SD. Statistically significant differences in amounts of damaged DNA were determined using LSD ANOVA test at a significance level of *p* ≤ 0.05. The significant difference between homogenous groups is depicted by a complete variation in the letters. The data are the results of four independent experiments.

Dimethyl sulfoxide (DMSO), which eliminates hydroxyl radicals and ozone [5,32], had an even higher scavenging effect which began to decrease at exposures higher than 20 s. Furthermore, no difference in scavenging activity was observed between samples treated with NTP for 2 to 20 s (Figure 4).

**Figure 5:**
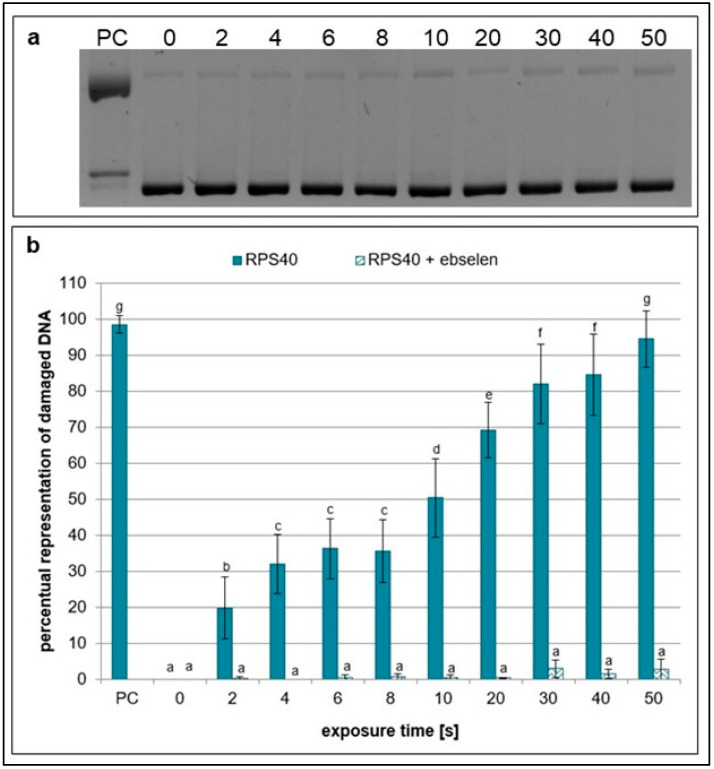
Representative gel (**a**) and graphical representation (**b**) of the percentage of damaged DNA in plasmid samples with ebselen (RPS40 + ebselen) and without a scavenger (RPS40) exposed to NTP for 2 to 50 s using RPS40 plasma source assessed by DNA topology assay. PC (positive control—0.125 mM FeSO_4_.7H_2_O), 0 (negative control—sample without NTP exposure). Damaged DNA represents the amount of both SSBs and DSBs. Data are presented as mean ± SD. Statistically significant differences in amounts of damaged DNA were determined using LSD ANOVA test at a significance level of *p* ≤ 0.05. The significant difference between homogenous groups is depicted by a complete variation in the letters. The data are the results of four independent experiments.

**Figure 6:**
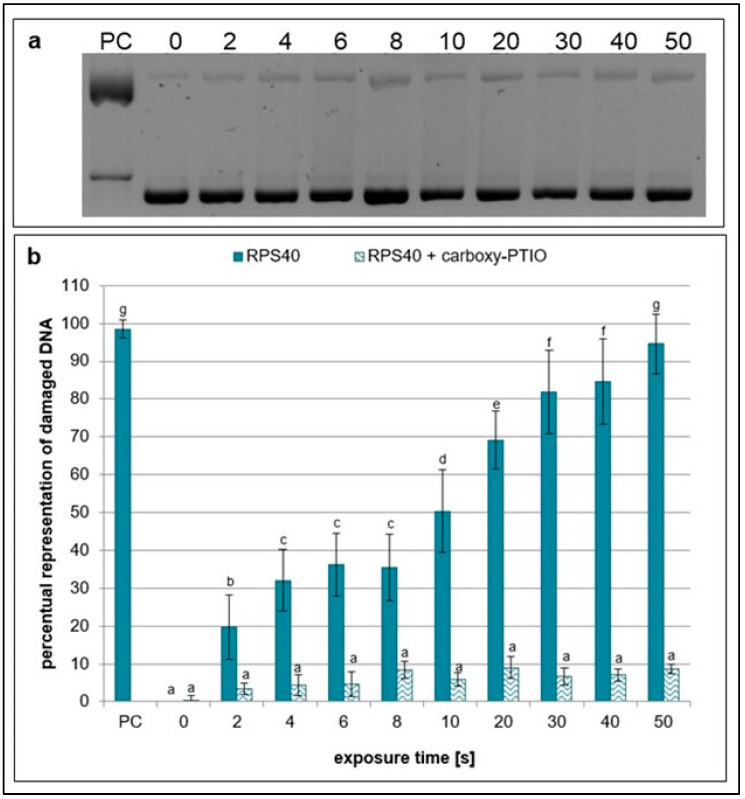
Representative gel (**a**) and graphical representation (**b**) of the percentage of damaged DNA in plasmid samples with carboxy-PTIO (RPS40 + carboxy-PTIO) and without a scavenger (RPS40) exposed to NTP for 2 to 50 s using RPS40 plasma source assessed by DNA topology assay. PC (positive control—0.125 mM FeSO_4_.7H_2_O), 0 (negative control—sample without NTP exposure). Damaged DNA represents the amount of both SSBs and DSBs. Data are presented as mean ± SD. Statistically significant differences in amounts of damaged DNA were determined using LSD ANOVA test at a significance level of *p* ≤ 0.05. The significant difference between homogenous groups is depicted by a complete variation in the letters. The data are the results of four independent experiments.

Ebselen, scavenger of nitric oxide [33,34], showed a high scavenging effect as almost no significant increase in damaged plasmid DNA at any treatment time was observed (Figure 5). The same effect was detected in samples containing carboxy-PTIO, which eliminates peroxynitrous acid, peroxynitrites, and hydrogen peroxide [30,35,36] (Figure 6). The results suggest that RPS40 plasma source predominantly produces hydrogen peroxide, peroxynitrites, peroxynitrous acid, nitric oxide, and its radicals which are well-eliminated by ebselen and carboxy-PTIO.

**Figure 7:**
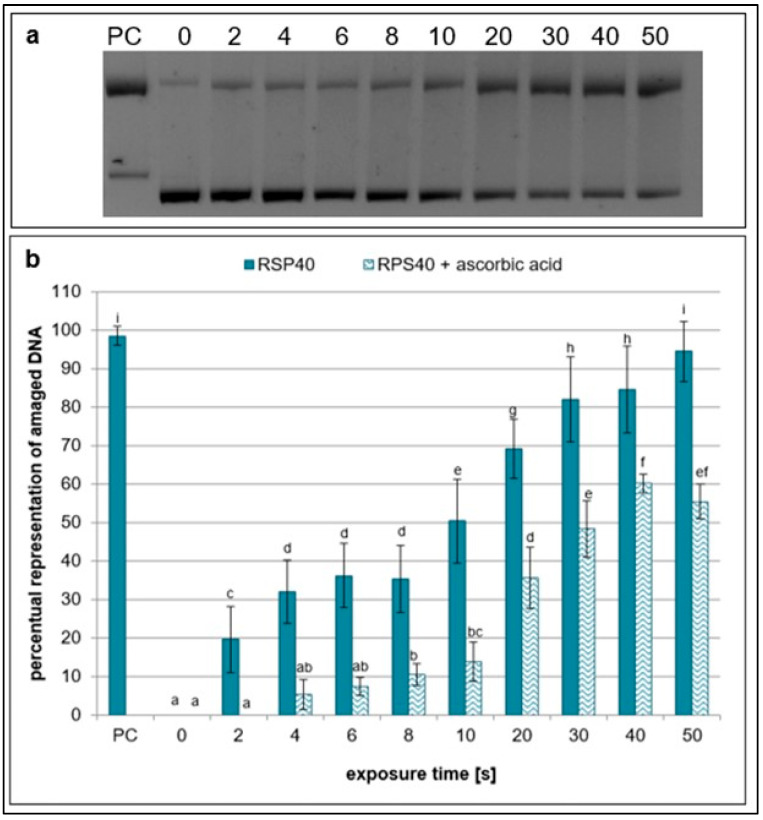
Representative gel (**a**) and graphical representation (**b**) of the percentage of damaged DNA in plasmid samples with ascorbic acid (RPS40 + ascorbic acid) and without a scavenger (RPS40) exposed to NTP for 2 to 50 s using RPS40 plasma source assessed by DNA topology assay. PC (positive control—0.125 mM FeSO_4_.7H_2_O), 0 (negative control—sample without NTP exposure). Damaged DNA represents the amount of both SSBs and DSBs. Data are presented as mean ± SD. Statistically significant differences in amounts of damaged DNA were determined using LSD ANOVA test at a significance level of *p* ≤ 0.05. The significant difference between homogenous groups is depicted by a complete variation in the letters. The data are the results of six independent experiments.

**Figure 8:**
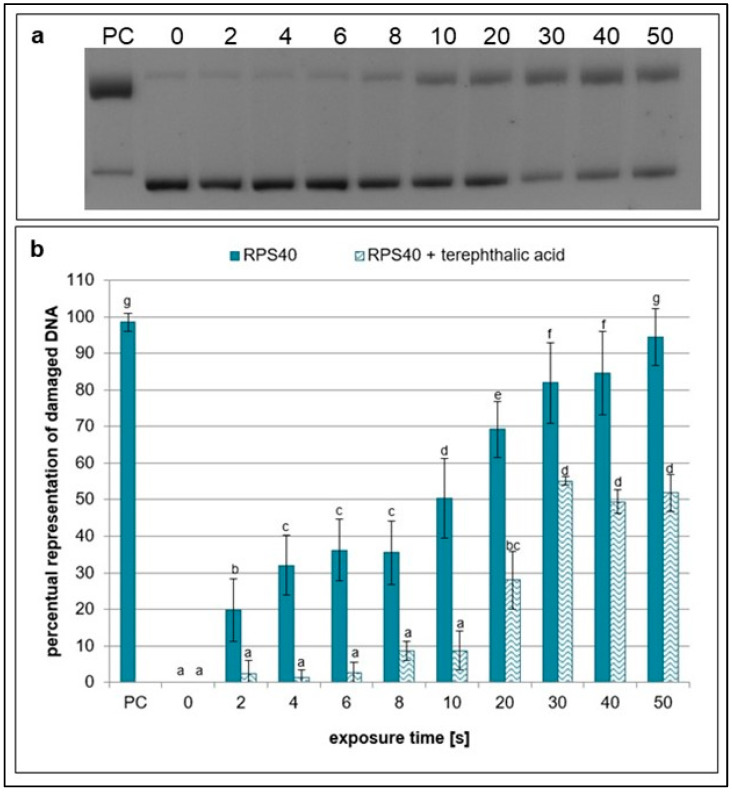
Representative gel (**a**) and graphical representation (**b**) of the percentage of damaged DNA in plasmid samples with terephthalic acid (RPS40 + terephthalic acid) and without a scavenger (RPS40) exposed to NTP for 2 to 50 s using RPS40 plasma source assessed by DNA topology assay. PC (positive control—0.125 mM FeSO_4_.7H_2_O), 0 (negative control—sample without NTP exposure). Damaged DNA represents the amount of both SSBs and DSBs. Data are presented as mean ± SD. Statistically significant differences in amounts of damaged DNA were determined using LSD ANOVA test at a significance level of *p* ≤ 0.05. The significant difference between homogenous groups is depicted by a complete variation in the letters. The data are the results of four independent experiments.

However, the scavenging activities of ascorbic (a scavenger of hydroxyl radicals and peroxynitrous acid) [29] and terephthalic acids (a scavenger of hydroxyl radicals) [37] were lower than those of the other scavengers. The amount of damaged plasmid DNA significantly increased with exposure time, and the difference in samples with and without the scavengers was the least significant (Figure 7 and Figure 8).

### 2.2. Effects of NTP on Concentration of Hydrogen Peroxide, Nitrates, and Nitrites

**Figure 9:**
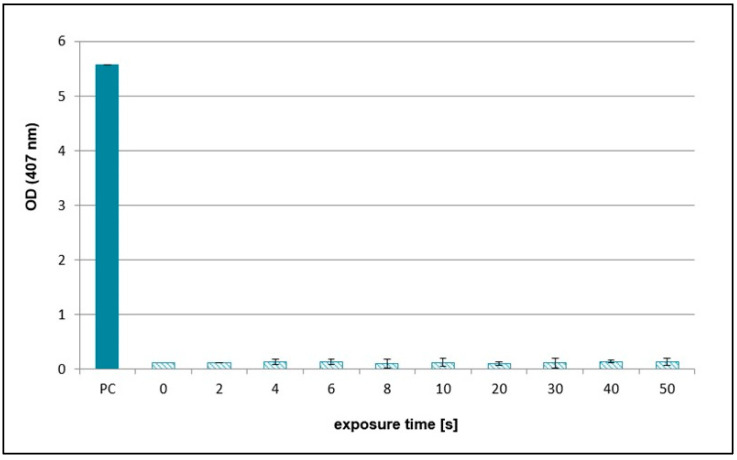
Graphical representation of hydrogen peroxide concentrations (absorbance) in reaction mixtures treated by NTP for 2 to 50 s using RPS40 plasma source. PC (positive control—25 mM hydrogen peroxide), 0 (negative control—sample without NTP treatment). Data are presented as mean ± SD. The data are the results of four independent experiments.

**Figure 10:**
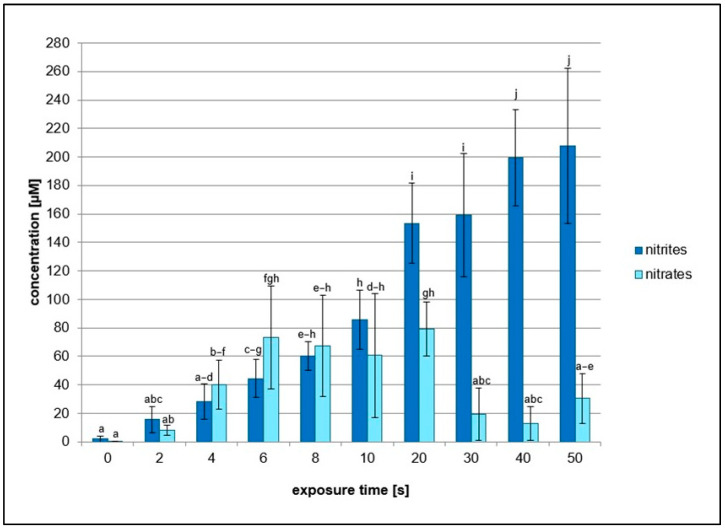
Graphical representation of nitrites and nitrates concentrations measured in reaction mixtures treated by NTP for 2 to 50 s using RPS40 plasma source. Data are presented as mean ± SD. Statistically significant differences in concentrations were determined using LSD ANOVA test at a significance level of *p* ≤ 0.05. The significant difference between homogenous groups is depicted by a complete variation in the letters. The data are the results of four independent experiments.

As the various scavengers had positive effects on the reduction in plasmid DNA SSB and DSB formation after NTP treatment probably due to the elimination of specific ROS and RNS, analyses of concentrations of nitrates, nitrites, and hydrogen peroxide were performed. The concentrations of hydrogen peroxide in the NTP-treated samples at different exposure times were the same as in the untreated sample, which was established as 0 (Figure 9). On the other hand, concentration of nitrites increased with exposure time. However, nitrates concentrations were lower compared to nitrites and their amount increased to 20 s treatment time and then decreased (Figure 10).

### 2.3. NTP Impact on pH and Evaporation

The diffusion of reactive species generated in NTP can cause change in pH value and evaporation of the water from the liquid surface. This was evident by the decreased pH levels at higher exposure times as well as the rate of water evaporation from the samples determined by the samples weight (Table 1).

### 2.4. Differential Scavenger Efficacy on Lymphocyte DNA

**Figure 11:**
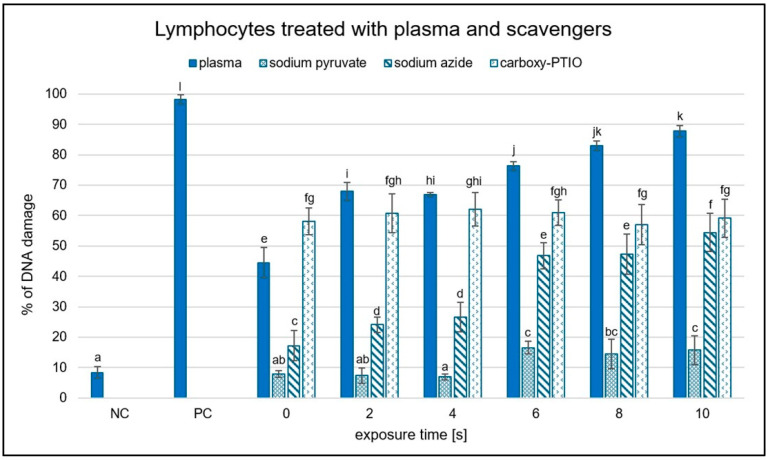
Graphical representation of the percentage of damaged DNA in lymphocytes treated with three selected scavengers and exposed to NTP for 2 to 10 s using RPS40 plasma source assessed by comet assay. NC (negative control—lymphocytes treated with phosphate buffer solution), PC (positive control—2.5 µM hydrogen peroxide), exposure time 0 (solvent control—lymphocytes treated with distilled water and scavenger solution). Data are presented as mean ± SD. Statistically significant differences in the percentage of DNA damage were determined using LSD ANOVA test at a significance level of *p* ≤ 0.05. The significant difference between homogenous groups is depicted by a complete variation in the letters. The data are the results of at least three independent experiments.

Following plasmid exposure to NTP, the potential of the selected reactive species scavengers to exert protective effects in a cellular environment was subsequently evaluated (Figure 11). Of the scavengers tested, only sodium pyruvate, sodium azide, and carboxy-PTIO could be applied to the samples, as the others required dissolution in pure DMSO. However, due to its nature as an organic solvent, DMSO dissolved the agarose block containing lymphocyte DNA and, at the required concentration, would lyse the cells and exert toxic effects, rendering those scavengers unsuitable for use in this system. Ascorbic acid was also excluded from the experiments due to its strong DNA-damaging effect at the concentration used in the DNA topology assay—even at 0 s of exposure—making it an inappropriate candidate (unpublished results).

Despite being limited to three scavengers, sodium pyruvate demonstrated the strongest protective effect, while carboxy-PTIO was the least effective. Sodium azide exhibited a moderate level of protection, showing consistently greater DNA preservation than carboxy-PTIO across all exposure times except at 10 s. Notably, carboxy-PTIO alone induced approximately 60% DNA damage, a value that remained consistent regardless of treatment duration. The scavenging efficacy of both sodium pyruvate and sodium azide decreased progressively with longer exposure times.

## 3. Discussion

Non-thermal plasma is an intensively studied phenomenon with many potential applications in biology. Due to its extensive practical applications, it is very important to understand its effects on living organisms and their components. Many laboratories are also studying the effect of plasma on DNA and its genotoxic potential, mainly on bacteria [38], plant [26,28], and animal cells [16,39], as well as normal and tumor cells [40,41]. However, in cells, DNA is protected by cellular and nuclear membranes and is continuously maintained by DNA repair mechanisms, which makes it difficult to evaluate the direct effects of plasma-generated species or species formed in water after plasma treatment. For this reason, plasmid DNA is advantageous as a model system. Using plasmid DNA allows us to assess the direct impact of plasma on DNA without the confounding effects of cellular repair processes or secondary damage. However, there are few studies that focus on the identification of species in plasma that can affect DNA molecules or that only investigate the direct effect of plasma on DNA itself in vitro. In our work, different scavengers of specific reactive oxygen and nitrogen species (RONS) were used for this purpose. The role of specific plasma-generated RONS in DNA damage induction was evaluated by analyzing the formation of distinct DNA forms in the presence or absence of individual scavengers.

**Figure 12:**
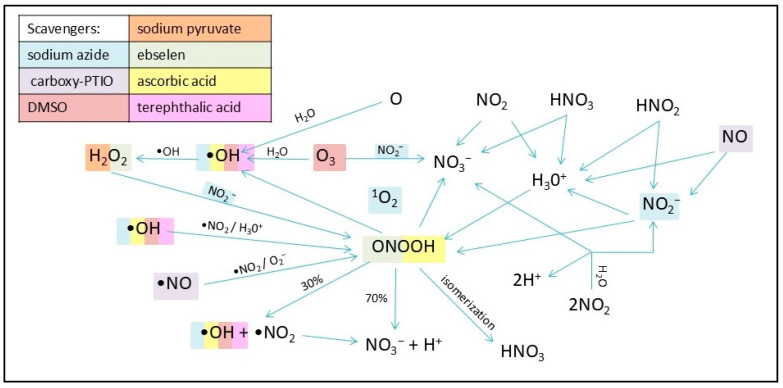
Schematic representation of chemical reactions processes probably taking place in PAW and the elimination effect of the scavenger used. The specific ROS and RNS that are eliminated by a given scavenger are color-correlated. Diffusion of more stable particles (H_2_O_2_, O_3_, NO_2_, HNO_2_, HNO_3_, NO) originating from plasma treatment causes generation of short-lived species in the water (OH and NO radicals, nitrites, nitrates). These short-lived species react with one another and with water molecules, which results in generation of secondary reactive species such as peroxynitrous acid, nitrites, nitrates, and hydroxyl radicals.

The plasma source RPS40 showed an increasing genotoxic potential with increasing exposure time in plasmid DNA. After 10 s, almost the entire plasmid DNA in the samples changed its topology due to the induced DNA damage. Various ROS are thought to induce SSBs more frequently, while DSBs are likely the result of two SSBs occurring in proximity on opposing DNA strands. Similar results were observed when helium plasma jet was used for a plasmid treatment [25,42,43,44]. Additionally, water evaporation from the liquid surface was observed as presented in Table 1. This may suggest gas–liquid interaction between reactive species created in plasma and water surface of the samples. Surface liquid is bombarded with reactive species, which causes dispersal of water molecules into the gas phase, and more reactive species are created (e.g., hydroxyl radicals, hydrogen peroxide, hydrogen radicals, and nitrogen oxides) [45]. It is well-known that plasma generates ROS and RNS, which are then dissolved in the treated solution. Our main goal was to determine the reactive species responsible for DNA damage formation. By applying seven different scavengers to the reaction mixtures, a decrease in plasmid DNA damage level in all samples was observed. Ebselen, carboxy-PTIO, and DMSO had the most notable scavenging activities out of all scavengers (Figure 4, Figure 5 and Figure 6). However, in the case of DMSO, its scavenging ability decreased after 20 s of exposure time (Figure 4). Based on these results, we propose that in our experimental conditions, most of the observed DNA damage could be caused by RNS such as peroxynitrites, peroxynitrous acid, nitric oxide, nitric oxide radicals, and ROS such as hydroxyl radicals, and ozone. It is likely that, due to the distance between the plasma source and the sample, long-lived particles such as nitrous acid, nitric acid, ozone, nitrogen dioxide, nitrogen oxide, and possibly hydrogen peroxide diffuse into the water containing plasmid DNA. These reactive species give rise to secondary reactive particles, primarily peroxynitrous acid, hydroxyl radicals, nitrites, and nitrates (Figure 12). DMSO and carboxy-PTIO showed strong scavenging activity against their respective ROS and RNS, including hydroxyl radicals, ozone, peroxynitrite, and hydrogen peroxide. Similarly, Ebselen displayed significant scavenging activity against nitrogen oxide and its radical, suggesting that these RNS contribute to the generation of additional reactive species in water and may also damage plasmid DNA. We propose that removing these specific species prevents the formation of secondary reactive species and, in turn, protects plasmid DNA from their damaging effects. Carboxy-PTIO exhibited a markedly different effect in lymphocytes, causing significantly higher DNA damage on its own compared to the negative control (Figure 11). Ascorbic acid induced an even higher level of DNA damage—substantially exceeding that of carboxy-PTIO—which can be attributed to its pro-oxidant behavior at high concentrations [46]. This pronounced cytotoxic effect led to its exclusion from further experiments. Finally, DMSO and any scavengers requiring it as a solvent could not be included in the comet assay due to technical limitations. Specifically, the concentration of DMSO required for dissolving scavengers, as used in plasmid DNA treatment, induced extensive DNA damage and would result in lymphocyte lysis and cell death.

Regarding hydrogen peroxide, sodium pyruvate significantly decreased the amount of damaged DNA, but compared to ebselen, its scavenging activity was much lower (Figure 2 and Figure 5). In lymphocytes, the amount of DNA damage after sodium pyruvate treatment was comparable to that of the negative control up to 4 s of exposure, and its protective effect was evident across all exposure times (Figure 11). The measured concentrations of hydrogen peroxide in the samples treated by NTP were the same as in the untreated control, suggesting that if some amount of hydrogen peroxide was present in the samples, its concentration had to be lower than 25 mM and even 10 µM based on the calibration curve data (Figure 9). When different authors treated water with various plasma sources, higher amounts of hydrogen peroxide were always detected [47,48,49]. However, Pandey et al. [50] used a similar type of plasma source to ours, and they also did not detect any increase of hydrogen peroxide concentrations. Nevertheless, our hypothesis is that a very low amount of hydrogen peroxide was present in our samples, as indicated by the sodium pyruvate scavenging activity (Figure 2), and that this molecule further contributes to reactions in plasma-treated water. Compared to the other studies mentioned above, our exposure times were much shorter, and our reaction mixture had smaller volume. Another reason for low hydrogen peroxide concentrations in the plasma-treated water could be low humidity in the ambient air. Wartel et al. [51] yielded similar results by treating the water with gliding arc plasma source using dry air as a working gas. They concluded that because of the very low humidity in the working gas, there was a low formation of hydroxyl radicals in the plasma, which resulted in lower formation of hydrogen peroxide. It is assumed that a substantial amount of hydroxyl radicals may be present in the reaction mixtures, given the significant scavenging activity observed for ascorbic acid, terephthalic acid, sodium azide, and DMSO (Figure 3, Figure 4, Figure 7 and Figure 8). In the case of lymphocytes, only sodium azide from the previously mentioned scavengers could be used. It significantly reduced DNA damage following plasma treatment; however, its protective effect was lower after 4 s of exposure. It is possible that hydroxyl radicals’ formation can be a consequence of reactions of other ROS (ozone) and RNS (nitrous acid, nitric acid, nitrogen dioxide, and nitric oxide) generated by plasma and later dissolved in the treated liquid. Nitrous acid and nitric oxide produce peroxynitrous acid, which can then deprotonate into hydroxyl radical and radical of nitrogen dioxide or nitrate and hydrogen ion [52]. Therefore, it is proposed that DNA damage might not necessarily be caused by peroxynitrous acid itself, but rather by its reaction products. Other authors also agree on the prevalence of peroxynitrous acid in plasma-treated water and its importance in cytotoxic effects [30,53,54]. Another evidence for the presence of peroxynitrous acid in the plasma-treated samples may be a decrease in pH values at higher exposure times (Table 1). Production of peroxynitrous acid is propagated by acidic environment and its decomposition itself adds to the acidification of the water [54]. Besides hydroxyl radicals and peroxynitrous acid, nitrites and nitrates represent other RONS primarily responsible for DNA damage. Our results show that concentration of nitrites increased with exposure time and nitrates concentrations increased to 20 s treatment time and then decreased (Figure 10). The decrease in nitrate concentration after 20 s is likely because the reaction mixture was not directly exposed to plasma but instead primarily treated with plasma processed air. We hypothesize that this method of plasma treatment caused inconsistent formation of these reactive species in the reaction mixture. From the scavengers used in the experiments only sodium azide eliminates nitrites. By adding it to the reaction mixture, a significant decrease in DNA damage was observed up to 20 s, after which the level of DNA damage began to increase (Figure 3). Nitrites and nitrates are products of reactions occurring in plasma-treated water and their concentrations are therefore affected by other reactive species formed during plasma generation. Concentration of nitrites in plasma-treated solutions decreases over time due to reaction with ozone and hydrogen peroxide. However, nitrate concentration can increase with treatment time and after treatment as was previously shown [50,51,52]. It is possible that nitrites and nitrates are created in the water as the result of nitrous and nitric acid diffusion. Decay of these acids contributes to the acidification of the water (Table 1), which can in turn give support to the formation of peroxynitrite and peroxynitrous acid [54].

We acknowledge several limitations of this study, including the restricted sensitivity of the colorimetric assay for H_2_O_2_, the indirect nature of DNA damage detection methods, the limited applicability of certain scavengers in cellular assays due to their toxicity, and the use of a single cell type. Nevertheless, the systematic combination of plasmid and cellular models with selective scavengers provides robust mechanistic insights that lay the groundwork for more detailed investigations employing advanced analytical techniques and diverse biological systems.

We conclude that the main functional species responsible for the observed plasmid and lymphocyte DNA damage under our experimental conditions could be peroxynitrous acid, hydroxyl radicals, nitrites, and nitrates (Figure 12). Hydrogen peroxide may be present in the samples in very low concentrations and could be involved in the ongoing chemical reactions. We propose that rather long-lived species (nitric oxides, nitrous and nitric acids, ozone, and possibly hydrogen peroxide) diffuse from the plasma’s gas phase to the treated liquid. There they undergo various chemical reactions resulting in the formation of short-lived species, e.g., hydroxyl radical, peroxynitrites, nitrites, and nitrates. Concentrations of ROS and RNS in the plasma-treated water increase with exposure time as scavenger’s elimination activities decrease. The decrease in pH value indicates the presence of acids in the plasma-treated samples such as nitrous and nitric acids, which can be a source for nitrites, nitrates, and peroxynitrous acid at higher exposure times. The chemical composition of plasma-treated water is highly dependent on many factors, i.e., humidity, plasma source set up, working gas, and volume of the treated liquid. Therefore, this topic needs further investigation to create definitive protocols for the usage of plasma-treated solutions in general practice.

## 4. Materials and Methods

### 4.1. Plasma Treatment

**Figure 13:**
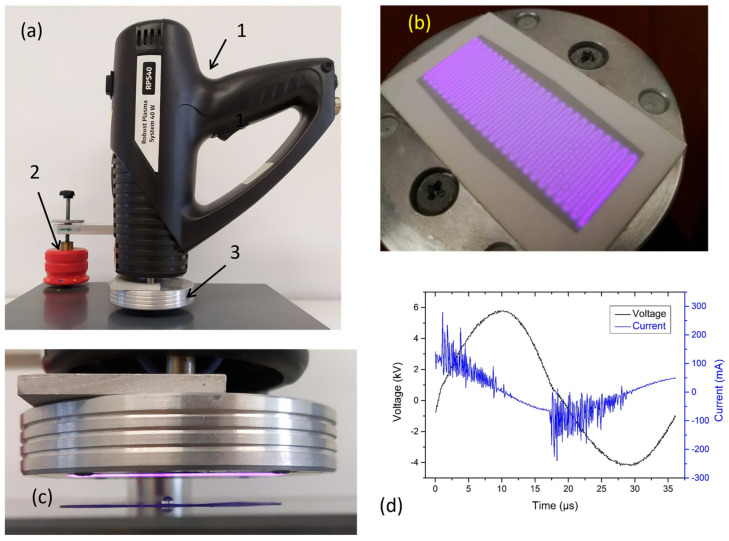
The photos illustrating the experimental set up, the general view of the handheld device RPS40 plasma system in the arrangement for the plasmid DNA treatment in the water, where 1—RPS40 plasma system body, 2—vertical holder with micrometrics displacement, 3—plasma head with electrode system (**a**), the view of the macroscopically homogeneous DCSBD plasma layer generated in ambient air (**b**), detail of the treatment process with sample position (**c**), and the time development of voltage and current waveforms in DCSBD generated in ambient air by RPS40 plasma system at input power 40 W (**d**).

As a source of non-thermal plasma for plasmid DNA treatment in the water, a small handheld portable ambient air plasma system manufactured by Roplass s.r.o., (Modrice, Czech Republic) and termed RPS40, was used in the experimental arrangement shown in Figure 13a. This plasma source is a miniaturized version of diffuse coplanar surface barrier discharge (DCSBD) with equally suitable properties of the generated plasma destined for the surface treatment of a wide range of materials. The RPS40 plasma source generates a diffuse, macroscopically homogeneous plasma layer on the surface of the alumina plate equipped with strip-like parallel electrodes which are covered by the thin transparent dielectric layer for mutual isolation of electrodes. The resulting plasma has dimensions of 50 mm × 20 mm with an effective thickness of 0.2–0.3 mm in ambient air (Figure 13b). During the operation, the electrode system is cooled down by the passive heat dissipation through aluminum housing (Figure 13c), which is simultaneously cooled by a fan. As already mentioned, the DCSBD plasma was successfully used for surface treatment of a broad spectrum of materials [55,56,57], also including the treatment of the plant seeds [58], and application for bio-decontamination [59,60,61] in recent years. In more detail, the RPS40 plasma source is described in [11] where the effect of non-thermal plasma generated on the structural and functional characteristics of human spermatozoa was studied.

The maximal uninterrupted operation time of RPS40 is limited up to 45 s. Subsequently, the plasma is automatically switched off, and the system is cooled down for 210 s. After this cooling down period, the RPS40 system can be used again. Due to the effective cooling of the electrode system, the temperature of the alumina plate is less than 60 °C for the maximum operation time. The electrical characteristics of RPS40 discharge system in terms of the voltage and current waveforms (Figure 13d) were measured by two high voltage probes Tektronix—P6015A,(Tektronix Inc., Beaverton, OR, USA) and by Pearson 4100 Current monitor probe (Pearson Electronics Inc, Palo Alto, CA, USA). Digital oscilloscope RIGOL DS1104 Z (Rigol Technologies EU GmbH, Gilching, Germany) was used for recording the signals from the probes. The real power supplied to the DCSBD was calculated according to the definition as a certain integral from the product of the instantaneous values of voltage and current during one period, divided by the period.

The plasma treatment of the pBR322 plasmid was performed by RPS40 mounted in a special vertical holder with micrometric displacement (see Figure 13a,c). The distance of the plasma source from the surface on which the reaction mixture was placed was set to 5 mm. The plasma was generated in the air at atmospheric pressure, at the input power of 40 W, and samples were exposed to the RPS40 plasma source from 2 to 50 s, with the negative and positive controls being the exception. Because the plasma device can operate only for a limited time without interruption, 40 s treatment was divided into two 20 s exposures with 1 min pause in between to allow the system to cool down. Similarly, the 50 s treatment was divided into 30 s and 20 s exposure.

Reaction mixtures (9 µL) of individual samples contained 6 µL of distilled water and 3 µL of pBR322 plasmid (75 ng of DNA, New England Biolabs, Ipswich, MA, USA). For positive control, 1 µL of 0.125 mM FeSO_4_.7H_2_O (Sigma-Aldrich, St. Louis, MO, USA) instead of 1 µL of water was used (positive control was not treated with NTP). To identify the possible ROS and RNS responsible for single- and double-strand DNA break formation, 1 µL of scavenger solution (Table 2) was added to the reaction mixture right before plasma treatment. The concentration of each scavenger was chosen according to the literature review to optimize scavenging efficiency while minimizing any potential damaging effects on plasmid DNA.

### 4.2. DNA Topology Assay

Prior to electrophoresis, 1 µL of 0.1 M phosphate buffer solution (1 M K_2_HPO_4_, 1 M KH_2_PO_4_, pH 6, both from Sigma-Aldrich) was added to each reaction mixture to stop further reactions of reactive species with plasmid DNA, and samples with 2 µL of loading dye (50% glycerol (Reachem, Bratislava, Slovakia), 1 mg bromophenol blue (Lachema, Brno, Czech Republic), 0.5 M EDTA (pH 8.0), 1 mg xylene cyanol (both from Sigma-Aldrich)) were loaded into a 1% agarose (Roth, Karlsruhe, Germany) gel with addition of 7.5 µL GelRed dye (0.0075%,Sigma Aldrich, St. Louis, MO, USA). The gel was then placed in an electrophoretic chamber filled with TBE solution (pH 8.3; 40 mM Tris base, 45 mM boric acid, 1 mM EDTA (all from Sigma-Aldrich)), and the electrophoresis was performed at 100 V for 90 min. Gel visualization was performed on UVP GelDoc-It2 Imaging Systems (Analytik Jena, Jena, Germany) with VisionWork Acquisition and Analysis software v. 8.20.17096.9551 (Analytik Jena). The undamaged plasmid DNA retains a supercoiled form and moves the fastest in the gel. A single-strand DNA break produces an open circular (OC) form that moves the slowest in the gel, and a double-strand DNA break produces a linear (LIN) form. The forms of DNA representing DNA damage in individual lanes were quantified from the band intensity using the ImageJ software (1.53c program, Wayne Rasband, National Institute of Health, Kensington, MD, USA). The data in graphs were normalized to negative control (NC) by subtracting the numerical merit of the band intensity of damaged DNA in NC from all samples. Overall damaged DNA was calculated in individual lanes by adding the band intensities of OC and LIN forms (single- and double-strand DNA breaks, respectively).

### 4.3. Measurement of Hydrogen Peroxide, Nitrites, and Nitrates Concentrations

The presence of hydrogen peroxide in reaction mixture without plasmid DNA was measured using a colorimetric method, in which hydrogen peroxide reacts with TiOSO_4_ to form pertitanic acid. This reaction is followed by color change to yellow [62]. After plasma treatment, 100 µL of reaction mixture (10 drops of 10 µL reaction mixture) was mixed with 10 µL of 60 mM sodium azide (Sigma-Aldrich). Subsequently, the samples were pipetted into 96-well plate and 50 μL of pure TiOSO_4_ solution (Sigma-Aldrich) was added to each sample. Absorbance was measured at 407 nm on a spectrophotometer Varioskan Flash Multimode Microplate Reader (Thermo Scientific, Waltham, MA, USA).

The presence of nitrites and nitrates in reaction mixture was measured using a colorimetric method based on the Griess reaction. Nitrite reacts with sulfanilamide under acidic conditions to form a diazonium ion, which is then coupled with N-(1-naphthyl) ethylenediamine to form a chromophoric azo product. This compound turns the solution into purple. Nitrates must first be enzymatically converted to nitrites by nitrate reductase and after subtraction of the nitrite concentration, the nitrate concentration is obtained [63,64]. The commercial Nitrite/Nitrate Assay kit (Sigma-Aldrich) was used for nitrite and nitrate detection according to the manufacturer’s instructions. The samples were then pipetted into 96-well plate and absorbance was measured at 540 nm on a spectrophotometer Varioskan Flash Multimode Microplate Reader (Thermo Scientific).

### 4.4. Measurement of pH Values and Estimation of Water Evaporation

The pH values were measured in plasma-treated samples with semiquantitative method using MQuant indicator strips (Sigma-Aldrich). To estimate the level of water evaporation from the treated reaction mixture (9 μL of water), the weight of the water droplets was measured on an analytical balance (Boeco Germany, Hamburg, Germany).

### 4.5. Comet Assay

To compare the results from DNA topology assay, we performed other experiments with cells using comet assay according to [65,66]. The study was conducted in full accordance with the principles of the Declaration of Helsinki. The experimental protocol was reviewed and approved by the Ethics Committee at Faculty of Natural Sciences, Comenius University Bratislava, and the ethical approval number is ECH 19043. Lymphocytes were isolated from peripheral blood using Histopaque gradient medium (Sigma Aldrich) and centrifugation (200× *g*, 5 min, 4 °C). The pellet was then mixed with a 1% low melting point agarose (Roth) dissolved in phosphate buffer solution (137 mM NaCl, 8 mM Na_2_HPO_4_, 2 mM KH_2_PO_4_ (all from CentralChem, Bratislava, Slovakia), 2.7 mM KCl (Sigma Aldrich)), and 100 µL of this mixture was mounted on a glass slide coated with 1% normal melting point agarose (Roth). Cover slips were placed on the slides and gel was set to solidify at 4 °C for 10 min. After the gel was solid, the cover slips were removed, and different scavenger solutions (Table 2) and distilled water were added to the samples. After that, all samples were incubated for 30 min at 37 °C. One group of samples was treated with distilled water (to keep the same conditions for treatment as with the plasmid) and the other was treated with scavenger solutions. Samples for the negative control were treated by phosphate buffer solution and positive control by 2.5 µM hydrogen peroxide (Sigma Aldrich). Positive control was incubated with hydrogen peroxide for only 5 min. After the incubation period, the samples were subjected to NTP treatment for 2, 4, 6, 8, and 10 s. Slides were then washed with phosphate buffer solution and placed in lysis solution (2.5 M NaCl (CentralChem), 100 mM Na_2_EDTA, 10 mM Tris-HCl, and 1% Triton X-100 (all three from Sigma Aldrich); pH 10) for 1 h at 4 °C. Once lysed, the samples were placed in the electrophoresis chamber and fresh electrophoretic solution (10 M NaOH, 0.2 M Na_2_EDTA all from (Sigma Aldrich); pH > 13) was poured and unwinding step took place for 15 min at 4 °C. Electrophoresis was performed at 25 V for 30 min, after which the samples were neutralized with phosphate buffer solution and water (5 min each). After drying, the slides were stained with ethidium bromide dye (20 μg/mL; Sigma Aldrich) and 100 random nucleoids were scored in each sample (from 0 to 4; 0 = undamaged, 4 = all DNA in the tail) using fluorescence microscope (OLYMPUS BX 51, Olympus Corporation, Tokyo, Japan) with green excitation filter (UMWIG3).

### 4.6. Statistical Analysis

The results were processed in Microsoft Excel and statistically analyzed with the Statgraphics Centurion XV v. 15.2.05 (StatPoint, Inc., Warrenton, VA, USA). To evaluate the obtained data, the statistical test ANOVA (one-way analysis of variance) was chosen and the differences in means were compared using the LSD test (test of the least significant difference). Significant differences between homogeneous groups are indicated by distinct lettering. Thus, if two groups share at least one letter, no significant difference between them was detected. In cases where the analysis system assigned more than three letters to a group, we simplified the notation by using an alphabetical range (e.g., a–d for abcd). The significance level was set at *p* ≤ 0.05. All experiments were performed at least three times.

## Figures and Tables

**Table 1:** pH value in the NTP treated samples and changes in weight of the samples after the treatment due to evaporation. Data are presented as mean ± SD and the results of three independent experiments. Statistically significant differences in concentrations were determined using LSD ANOVA test at a significance level of *p* ≤ 0.05. The significant difference between homogenous groups is depicted by a complete variation in the letters.

Exposure Times [s]	pH Value	Sample Weight [mg]
0	5 ^c^	8.67 ± 0.12 ^g^
2	5 ^c^	8.50 ± 0.10 ^fg^
4	5 ^c^	8.57 ± 0.12 ^ef^
6	5 ^c^	8.40 ± 0.10 ^de^
8	5 ^c^	8.37 ± 0.06 ^de^
10	5 ^c^	8.33 ± 0.06 ^d^
20	5 ^c^	8.17 ± 0.06 ^c^
30	4 ^b^	8.17 ± 0.06 ^c^
40	3 ^a^	7.77 ± 0.06 ^b^
50	3 ^a^	7.57 ± 0.06 ^a^

**Table 2:** Scavenger solutions used in the experiment and specific ROS/RNS they eliminate.

Scavenger	ROS/RNS	Concentration *	Solvent	References
sodium pyruvate	H_2_O_2_	100 mM	H_2_O	[29]
sodium azide	^1^O_2_, •OH, NO_2_^−^	50 mM	H_2_O	[29,30,31]
carboxy-PTIO	ONOO^−^, ONOOH, H_2_O_2_	1 mM	H_2_O	[30,35,36]
ebselen	NO, •NO	10 mM	DMSO	[33,34]
ascorbic acid	ONOOH, •OH	20 mM	H_2_O	[29]
dimethyl sulfoxide	•OH, O_3_	99%	-	[5,32]
terephthalic acid	•OH	1 mM	DMSO	[37]

* Each scavenger (all were provided from Sigma-Aldrich) was added to the reaction mixture before treatment and was therefore diluted at 1:8. Concentrations of scavengers (presented in stock solutions) were selected based on the literature review to ensure their high scavenging ability while not causing plasmid DNA damage at the same time. The effect of scavengers on the plasmid is depicted in the graphs as exposure time 0 s.

## Data Availability

The original contributions presented in the study are included in the article; further inquiries can be directed at the corresponding author.
